# Nucleoside Analogs: A Review of Its Source and Separation Processes

**DOI:** 10.3390/molecules28207043

**Published:** 2023-10-12

**Authors:** Pan Wang, Tao Cheng, Jianming Pan

**Affiliations:** School of Chemistry and Chemical Engineering, Jiangsu University, Zhenjiang 212013, China; wangp@ujs.edu.cn (P.W.); 2212212006@stmail.ujs.edu.cn (T.C.)

**Keywords:** nucleoside analogs, separation methods, molecular imprinting techniques, selective separation and purification

## Abstract

Nucleoside analogs play a crucial role in the production of high-value antitumor and antimicrobial drugs. Currently, nucleoside analogs are mainly obtained through nucleic acid degradation, chemical synthesis, and biotransformation. However, these methods face several challenges, such as low concentration of the main product, the presence of complex matrices, and the generation of numerous by-products that significantly limit the development of new drugs and their pharmacological studies. Therefore, this work aims to summarize the universal separation methods of nucleoside analogs, including crystallization, high-performance liquid chromatography (HPLC), column chromatography, solvent extraction, and adsorption. The review also explores the application of molecular imprinting techniques (MITs) in enhancing the identification of the separation process. It compares existing studies reported on adsorbents of molecularly imprinted polymers (MIPs) for the separation of nucleoside analogs. The development of new methods for selective separation and purification of nucleosides is vital to improving the efficiency and quality of nucleoside production. It enables us to obtain nucleoside products that are essential for the development of antitumor and antiviral drugs. Additionally, these methods possess immense potential in the prevention and control of serious diseases, offering significant economic, social, and scientific benefits to the fields of environment, biomedical research, and clinical therapeutics.

## 1. Introduction

**Figure 1:**
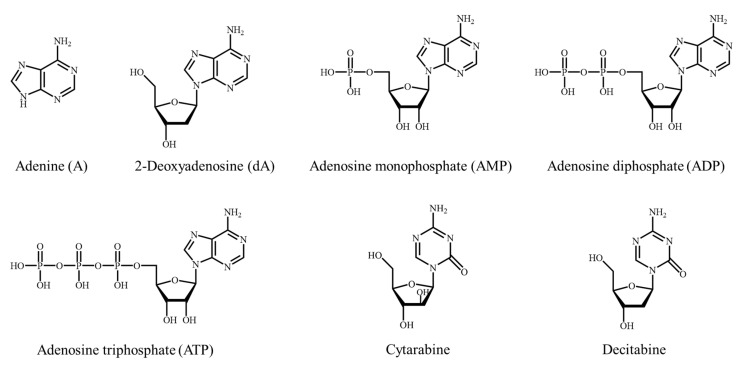
Molecular formula of A, dA, AMP, ADP, ATP, Cytarabine, and Decitabine.

Nucleoside analogs are a class of water-soluble components with a wide range of physiological activities, which play an essential role in various biological processes. This class of compounds can be divided into three categories: bases, nucleosides, and nucleotides [1,2,3]. The bases, including adenine, guanine, cytosine, thymine, and uracil, are the building blocks of nucleic acids [4,5,6]. Derivatives can be classified into purines and pyrimidines, which are important components of nucleotides [7,8]. Nucleotides are compounds composed of a combination of a base and ribose or deoxyribose in a glycosidic bond. The purine bases include adenine and guanine, while the pyrimidine bases include cytosine, thymine, and uracil [9,10,11]. By understanding the properties and functions of purines and pyrimidines, scientists can gain insights into the fundamental mechanisms of life. Nucleotides are compounds formed by a phosphodiester bond and the 5-hydroxyl or 3-hydroxyl group of a nucleoside. Phosphates in nucleotides have three molecular forms, as shown in Figure 1 [12,13,14]. Nucleotides are the basic building blocks of ribonucleic acid (RNA) and deoxyribonucleic acid (DNA), which have a variety of significant biological functions. They are involved in the storage, copying, and transformation of genetic information in almost all living cells. Furthermore, they play a crucial role in the functioning of organisms and the inheritance of traits [1,15]. Therefore, nucleotides are vital raw materials for biochemical drugs and genetic engineering research and are also important intermediates for the production of antibacterial, antitumor, antiviral, antifungal, and immunomodulatory nucleoside analogs [16,17,18,19,20].

Since the 1940s, there has been ongoing research on the structural and pharmacological studies of nucleoside analogs. For instance, cytarabine was first approved by the U.S. Food and Drug Administration (FDA) in the 1960s for the treatment of acute myeloblastic leukemia. In addition to cytarabine, a wide range of nucleoside analogs have been synthesized and tested for their efficacy in clinical cancer treatment [21,22]. Azacitidine and decitabine, approved in 2004 and 2006, respectively, were initially developed as demethylating agents. Meanwhile, besides their demethylating properties, they also exhibited significant antiproliferative activity against cancer cells (Figure 1) [23,24]. In addition, a massive number of nucleoside analogs are under development, for example, the research conducted by Christoph Kollmann’s group, who synthesized 4′/5′-spirocyclopropanated uridine [25] and C4′-methylated uridine [26], studying their pharmacological properties against various respiratory viruses. Gertrude B. Elion’s groups [27,28] synthesized antiviral nucleoside analogs that acyclovir based on 6-mercaptopurine. Chris Meier’s groups [29] studied the development of anti-HIV-active nucleoside triphosphate prodrugs and evaluated their activity in antiviral tests. Meanwhile, in recent years, nucleoside analogs have been widely used as essential components in antibiotics to regulate the physiological effects of the immune, nervous, metabolic, liver, and cardiovascular systems, thereby improving human health support and preventing diseases [30,31,32,33]. Therefore, the development and research of nucleoside analogs have great economic, social, and scientific significance in the fields of biomedicine and human life and health.

## 2. The Sources of Nucleoside Analogs

Nucleoside analogs are widely used in antibiotics, antibacterial, antitumor, antiviral, antifungal, and immunomodulatory drugs [34,35,36]. There are three main sources of nucleoside analogs, which differ according to their preparation method, including the degradation of nucleic acid, chemical synthesis, and biotransformation [37,38,39].

### 2.1. Degradation of Nucleic Acid

**Figure 2:**
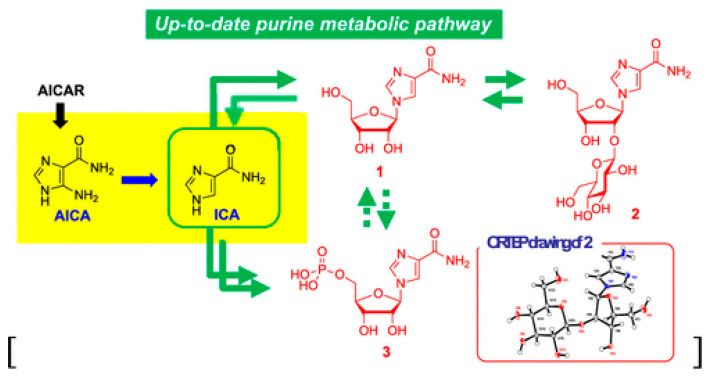
Novel purine metabolic pathway in rice [37].

The nucleic acid degradation method is mainly used to extract nucleic acids from various sources, including natural products such as Cordyceps sinensis, Ganoderma lucidum, rice [40,41,42], animal organs like liver, spleen, and thymus [43,44,45], as well as microorganisms such as yeast, brandy wine molds, and antimicrobial molds [46,47,48]. Nuclease enzymes are then employed to degrade ribonucleic acid and generate nucleotides. Furthermore, since the 1960s, most researchers have been conducting experiments on using nuclease to degrade RNA and produce mononucleotides. For instance, Patrick S. Fier groups [49] synthesized the antiviral drug molnupiravir for COVID-19 by studying the degradation behavior of different nucleoside phosphatases. Hirokazu Kawagishi groups [37] synthesized nucleoside products from rice by adjusting the inhibitory activity of ribonuclease and determined their structures using single-crystal X-ray diffraction techniques and spectral analyses (Figure 2). Wu groups [40] discovered two types of nucleosides and three types of sterols from the selected 90% methanol(aq) fraction of C. militaris fruiting bodies using the bioactivity degradation method. They discussed the inhibitory and anti-inflammatory activities of the two obtained nucleosides—cordycerebroside A(1) and glucocerebroside (3). Lindner groups [16] successfully generated 16 nucleosides and nucleobases from both natural and cultivated Ganoderma lucidum. They achieved efficient separation of these products by varying the chromatographic conditions and optimizing the system. However, these methods often have some disadvantages. Firstly, the production time is usually long, taking more than two weeks from raw material preparation to product manufacturing. Furthermore, the complexity of the product matrix generated involves the simultaneous presence of many (deoxy) nucleotides, making the separation process of the target nucleoside compounds challenging. Moreover, the scarcity and declining quality of the ideal raw nucleotide material, combined with the intricate pre-processing procedures, make it unsuitable for large-scale production of nucleoside compounds [50,51,52].

### 2.2. Chemical Synthesis

**Figure 3:**
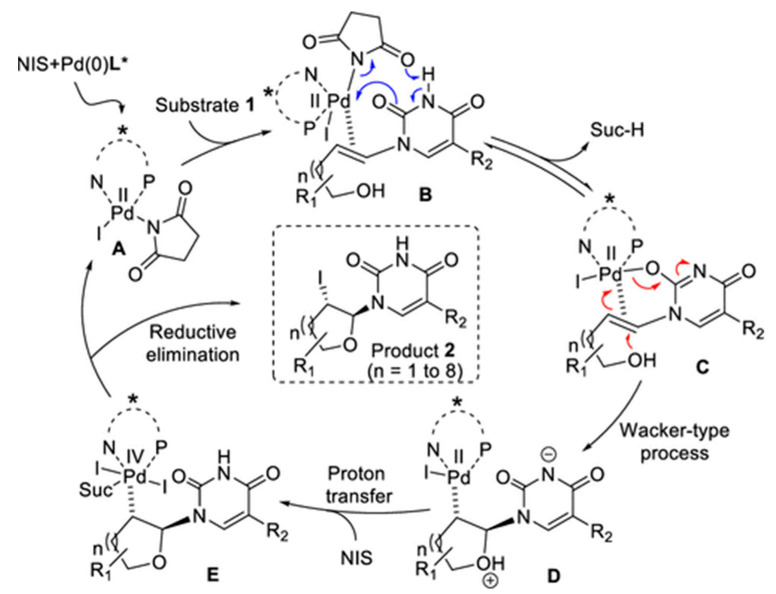
Reaction mechanism by chemical synthesis [54].

The molecular structure of nucleoside analogs is relatively simple, which makes them easy to produce through chemical synthesis. They have aroused increasing interest in recent years, leading to numerous studies on their preparation using chemical synthesis. Yu groups [53] used a linear synthesis strategy to synthesize the nucleoside antibiotics A201A and A-94964 by designing modular synthesis routes. Ehesan USharif et al. [54] synthesized a variety of iodine atom-functionalized pyrimidine nucleoside analogs via palladium (Pd) catalysis (Figure 3). John D. Sutherland et al. [55] prepared adenine nucleoside analogs through a multi-step chemical reaction of imidazoles. Chemical synthesis is widely used for the quick and efficient synthesis of unnatural nucleoside analogs [56,57]. Michal Hocek groups [38] successfully synthesized a series of 7-[2-(alkyl- or arylsulfanyl)-ethyl]-7-deaza-2′-deoxyadenosines by phosphorylation. Additionally, the modified phosphates were prepared by adding thiol to 7-vinyl-7-deaza-dATP. Piet Herdewijn groups [58] employed palladium-catalyzed cross-coupling chemistry for the synthesis of C-7-alkynylated and arylated pyrrolotriazine C-Ribonucleosides. Masayuki Inoue groups [59] have developed a novel method of coupling multiply-hydroxylated aldehydes with α-alkoxyacyl tellurides in order to synthesize various nucleoside antibiotics. Gustavo Moura-Letts groups [60] demonstrated high efficiency in synthesizing nucleoside carbacycles through sequential dipolar cycloaddition and reductive cleavage of enals and hydroxylamines. Guo et al. [61] used enantioselective [3+2] cycloaddition of α-nucleobase substituted acrylates to prepare chiral carbocyclic nucleoside analogs, including purine, uracil, and thymine derivatives, with high quaternary. Meanwhile, considering that glycosylation is an extremely important biological process in the synthesis of nucleoside analogs by chemical methods, Downey, A. M. groups [62,63] studied the mechanism of the occurrence of glycosylation reaction through selective activation of the anomeric center on the glycosyl donor and direct glycosylation of nucleobases methods, and verified the feasibility of the strategy of glycosylating protecting group-free strategies for the efficient synthesis of nucleoside analogs. This method provides a straightforward approach to the synthesis of these analogs. However, the reaction reagents often involved in chemical synthesis are mostly chemically toxic, which poses a threat to the production of nucleoside analogs and does not meet the requirements of green environmental protection [64,65]. At the same time, the target nucleoside analogs must be activated. This activation involves complex steps, including the protection and deprotection of specific groups during the synthesis process. However, these steps can lead to the production of difficult-to-separate enantiomers.

### 2.3. Biotransformation

**Figure 4:**
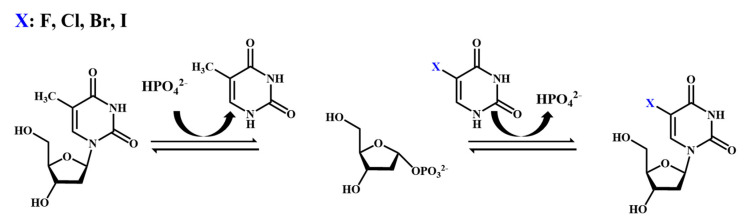
Two-step, one-pot enzymatic reaction for the synthesis of halogenated pyrimidine nucleosides using thymidine as a sugar donor [70].

The biotransformation method utilizes naturally occurring enzymes in organisms to catalyze unit substitution or modification reactions, leading to the synthesis of new nucleoside analogs based on existing fermentation substrates [66,67]. Takashi Tsuji et al. [68] proposed a biotransformation method for producing 2′-deoxyadenosine, which uses 2′-deoxythymidine as a ribose group donor and adenine as a base donor. Ying groups [69] synthesized adenosine triphosphate using the yeast Saccharomyces cerevisiae while studying the various factors that influence the yield. Francesca Paradisi et al. [70] successfully synthesized four nucleoside analogs: 5-fluoro-2′-deoxyuridine, 5-chloro-2′-deoxyuridine, 5-bromo-2′-deoxyuridine, and 5-iodo-2′-deoxyuridine, using a newly discovered thymidine phosphorylase, and the conversion rate reached 90% at a concentration of 10 mM (Figure 4). Enrica Calleri synthesized [71] adenine nucleosides using bioreactors based on two sequential nucleoside phosphorylases: uridine phosphorylase (CpUP) and purine nucleoside phosphorylase (AhPNP). Compared with nucleic acid degradation and chemical synthesis, the biotransformation method is simple, uses fewer organic reagents, has gentle conditions, has lower costs, and is environmentally friendly [72,73]. Therefore, the biotransformation method is a common approach for producing nucleoside analogs. However, the concentration of substrates and products in the biotransformation solution during biosynthesis is usually very low, and the impurities, such as by-products, are high, which seriously restricts the pharmaceutical research and application development of high-quality nucleoside analogs.

## 3. Progress in the Separation of Nucleoside Analogs

It was found that nucleoside analogs synthesized using the three methods still have some common problems. For instance, the main product has a low concentration [74], the matrix is complex [75], and there are numerous by-products [76]. Therefore, finding a suitable method for the separation and enrichment of nucleoside analogs from the products becomes an emergency. There are currently many methods for the separation of nucleoside analogs, such as crystallization, high-performance liquid chromatography (HPLC), column chromatography, solvent extraction, and adsorption [77,78,79,80,81]. Additionally, these methods can effectively separate and enrich the desired products when used. Table 1 summarizes the advantages and disadvantages of several separation methods in Section 3.1, Section 3.2, Section 3.3, Section 3.4 and Section 3.5.

### 3.1. Crystallization

Crystallization is a technique for separating different components in a mixture by inducing a state of super-saturation in a solution. Various methods can be employed to achieve this state, including evaporation, cooling, vacuum cooling, solvent precipitation, salting out, and reaction crystallization [82,83,84]. Crystallization methods have been widely utilized by researchers to separate nucleoside analogs from crude products. For instance, organic solvents like ethanol or acetone can be added to reduce the solubility of adenosine 5′-monophosphate in the solvent, leading to the precipitation of the solute [94]. Ahmed M. Ibraheem groups [95] synthesized 2-oxonicotinonitriles and 2-oxonicotinonitrile and nucleoside analogs based on 2-oxonicotinonitrile, which showed good anti-SARS-CoV and anti-influenza A (H_5_N_1_) activities. Andrzej Okruszek’s group [96] achieved a specific separation of diastereomers by fractionally crystallizing suitable (aphenylethylamino) phosphoramidate precursors into individual diastereomers. The use of nucleoside compounds of phosphate (nucleoside-5′-phosphates) with barium and mercury ions to form a precipitate of heavy metal salts, which is precipitated from the solution, achieves the separation purpose [97]. Although the crystallization method for separating nucleosides is an affordable and easily industrialized process, its application often involves the use of toxic solvents or heavy metal salts, which means it can easily cause environmental pollution and result in high energy consumption. Additionally, the extensive scope of auxiliary equipment is unsuitable for handling products of nucleoside analogs in low concentrations.

### 3.2. High-Performance Liquid Chromatography 

**Figure 5:**
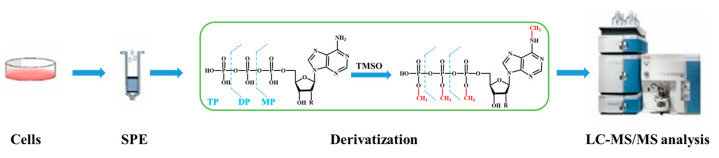
Procedure overview. Ribonucleotides (RNs) and deoxyribonucleotides (dRNs) of the intracellular via LC-MS/MS [101].

HPLC is a crucial method for modern separation and analysis. The components are dispersed in the mobile phase, which passes through the stationary phase. Based on the size and strength of the interaction between the stationary phase and each component (such as hydrogen bonding, electrostatic interaction, exclusion, etc.), the retention time in the stationary phase varies sequentially, achieving the separation of different components [85,86]. Combined with the principle of HPLC, many researchers have already used HPLC as an important tool for the quantitative analysis of nucleoside analogs from complex mixtures. Irene Suárez-Marina groups [98] used HPLC to quantitatively analyze nucleoside analogs in complex mixtures based on their different retention times and peak areas. They also employed mass spectrometry in an even better fashion to characterize the structure of synthesized nucleoside analogs. Thomas Carell groups [99] utilized ultra-high performance liquid chromatography (UHPLC) and triple quadrupole mass spectrometry (QQQ-MS) to accurately quantify DNA modifications within a short time (14 min per sample). Furthermore, their quantitative method allows for rapid, ultrasensitive (low concentration range), and highly reproducible measurement of various nucleotides. C. McGuigan groups [100] compared the advantages and disadvantages of HPLC and crystallization methods for separating nucleoside analogs. They also developed a new catalytic system to enhance the diastereoselectivity and yield in HPLC separation, aiming to improve the identification of target nucleosides. Zhang groups [101] have succeeded in identifying and separating endogenous nucleotides, nucleoside analogs, and their metabolites in complex samples by introducing methyl groups into the nucleotides. This modification increases the retention time of the molecules on HPLC columns (Figure 5). The researchers also employed a simple reversed-phase chromatographic condition to avoid contamination and ion suppression in mass spectrometry analyses induced by the ion-pairing reagents. HPLC offers the advantages of swift analysis and high sensitivity. However, its implementation in industrial applications is often hindered by the stringent requirements for separation conditions and high operating costs [102,103,104]. Moreover, the complexity of the method may lead to difficulties in practicality.

### 3.3. Column Chromatography

In column chromatography, the solid phase is installed in the column. Nucleoside compounds are contained in the mobile phase and flow from top to bottom through the stationary phase. The stationary phase has an interaction force that adsorbs the nucleoside compounds [87,88]. When the eluent flows through the stationary phase, different nucleoside compounds and the stationary phase have different forces, and the downward elution rate is also different to enrich different nucleoside analogs [105,106]. Column chromatography is commonly used to separate and enrich desired compounds from complex samples [107]. Recently, researchers have reported the successful separation and enrichment of nucleosides using column chromatography. Robert A. Keyzers groups [108] used HP20 and HP20ss reversed-phase column chromatography (PSDVB) to separate and enrich two nucleoside compounds from a mixture. Zhang groups [109] employed petroleum ether, ethyl acetate, and n-BuOH as eluents to separate nucleosides from a mixture based on their polarity difference. Piet Herdewijn groups [106] synthesized nucleoside analogs with phosphonate functional groups by chemical synthesis, specifically pentopyranoside nucleosides. They performed identification separations using silica gel column chromatography with different ratios and mobile phases. To enhance discriminatory separation and specifically capture the target nucleosides, Cao groups [110] used boronate to functionalize silica gel, preparing a high-affinity monolith column. The separation of nucleosides by column chromatography using the difference in polarity has been achieved. Column chromatography has a high separation efficiency and simple operation, but the use of an organic mobile phase can lead to environmental pollution.

### 3.4. Solvent Extraction Method

Solvent extraction is a process used to separate and purify solutes. It involves adding a solution containing the solute to another solvent, which completely transfers the solute to the new solvent [89,90]. Karl W.K. Tsim groups [111] extracted natural nucleosides from cordyceps sinensis using a pressurized solvent (methanol) and determined the content of nucleosides and their bases in combination with high-performance liquid chromatography (HPLC). Li groups [112] used various solvents, including those under pressure, boiling water, and ambient conditions, in combination with HPLC to determine the content of the five nucleosides (adenosine, guanosine, inosine, uridine, and cordycepin) in the extracted Cordyceps. Shim groups [113] extracted butterbur grown using three aqueous solvents at three different pH conditions. They then examined the extraction efficiency by utilizing the ultra-performance liquid chromatography-tandem mass spectrometry (UPLC-MS/MS) technique. This method has also been successfully applied in the study of antibiotic resistance gene development in humans and the environment. Ali Sefoddin et al. [80] used subcritical water instead of conventional extraction solvents to extract natural nucleoside components from abalone. The conventional extraction method often introduces more organic solvents, which can easily cause environmental pollution. Additionally, the conventional method lacks selectivity for nucleoside analogs with similar structures, which makes molecular identification impossible.

### 3.5. Adsorption

In the adsorption method, the target molecules are dissolved in the liquid phase, and then the adsorbent is dispersed in the liquid phase. The target molecules and the adsorbent connect through special forces (hydrogen bond, electrostatic effect, chemical bond, etc.), while the other substances are not adsorbed. Then, the captured target molecules are eluted with a small amount of eluent to achieve the purpose of adsorption separation and enrichment [91,92,93]. Some nucleoside analogs possess unique properties due to the presence of special functional groups, such as those with cis-dihydroxyl, phosphate, and base functional groups. Selective adsorption and separation of these compounds can be achieved by utilizing the affinity of specific functional groups for nucleoside analogs and the different chemical properties of the target molecules [114]. For example, selective separation can be achieved by analyzing the differences in specific functional groups, solubility, and light sensitivity of guanine and guanosine bands and preparing adsorbents with high identification [115,116]. There have been extensive studies on the separation of nucleoside analogs using the adsorption method. Ariga Katsuhiko groups [117] separated and purified nucleosides (adenosine, guanosine, and thymidine) using carbon nanocage, and they observed significant selectivity between purine-base and pyrimidine-base nucleosides by using carbon nanocage. Deniz Aktaş Uygun groups [118] developed new boronate affinity nanoparticles using a surfactant-free emulsion polymerization technique and then functionalizing them with phenylboronic acid to adsorb nucleosides. Liu groups [119] prepared an organic–inorganic composite cryogel with a three-dimensional hierarchical meso- and macroporous structure by freezing. This organic–inorganic low-temperature gel composite can effectively adjust the pore size with a large specific surface area, and the mesopores and macropores on the material provide enough reaction sites to effectively enhance the mass transfer efficiency. Therefore, it was used as an adsorbent for solid-phase extraction, for modifying boric acid sites, and for analyzing nucleosides in spiked human serum. Zhang groups [120] synthesized a boronate affinity material by grafting boronic acid groups onto attapulgite, which is a fibrous aluminum-magnesium silicate. This material can selectively capture cis-diols, such as nucleosides, and it has been applied to the selective extraction of nucleosides from human urine.

**Figure 6:**
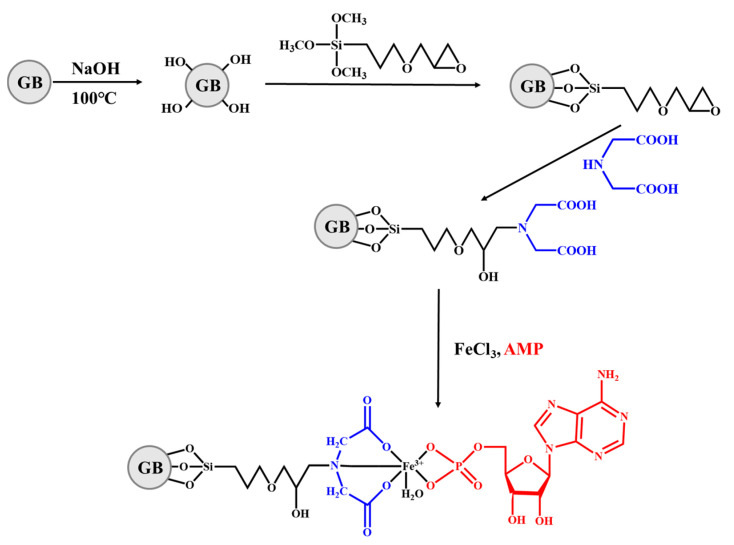
Synthesis route of MIP-NPs [125].

**Figure 7:**
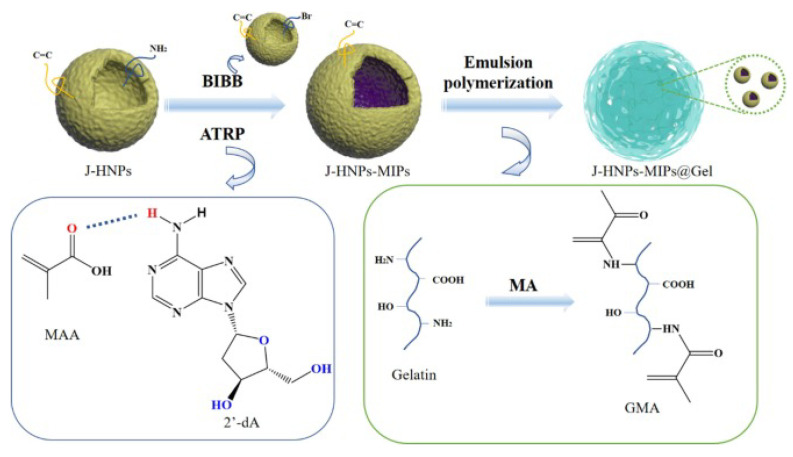
Synthesis route of composite hydrogel adsorbent encapsulating imprinted hollow mesoporous nanoparticles (MMHSG) [126].

Although the design of synthetic adsorbents based on the special functional groups of target molecules can effectively separate nucleosides, there is still a need for further improvement in the selectivity of conventional adsorbents for nucleosides with rich structures and similar functional groups. Molecular Imprinting Technology (MIT) is a method that mimics the principles of specific binding, similar to how enzymes bind to substrates or antigens bind to antibodies, in order to achieve strong and specific identification of particular targets [121,122]. MIT utilizes the target molecule as a template and selects appropriate functional monomers to synthesize polymers known as Molecularly Imprinted Polymers (MIPs), which exhibit specific recognition of the target molecule [123,124]. Nowadays, some researchers have proposed the use of MIPs as adsorbents for the specific separation and recognition of nucleoside analogs. Tse Sum Bui groups [125] reported the preparation of molecularly imprinted polymers for adenosine monophosphate (AMP) through the solid-phase synthesis method, which is shown in Figure 6. The experimental results of selective adsorption studies demonstrated that the molecularly imprinted polymers exhibit high binding capacity and selectivity for the target molecules AMP. Pan groups [126] used the electron-activated regenerative atom transfer radical polymerization (ARGET-ATRP) technique to graft dA-MIPs of dA onto the inner and outer surfaces of hollow mesoporous silica particles, resulting in the formation of imprinted nanoparticles MMHS. Additionally, a single microgel encapsulated-emulsion template method was employed to prepare a composite gel adsorbent (MMHSG) (Figure 7) for selective extraction of dA, exhibiting an adsorption capacity of 20.22 μmol g^−1^. Luigi A Agrofoglio groups [127] successfully prepared a highly cross-linked polymer imprinted with monophosphate (AMP) by utilizing a phosphate functional group. This synthesized imprinted polymer was then packaged in an SPE column. The results of the selectivity analysis showed that the imprinted polymer exhibited good specificity not only for AMP but also for other nucleotides.

**Figure 8:**
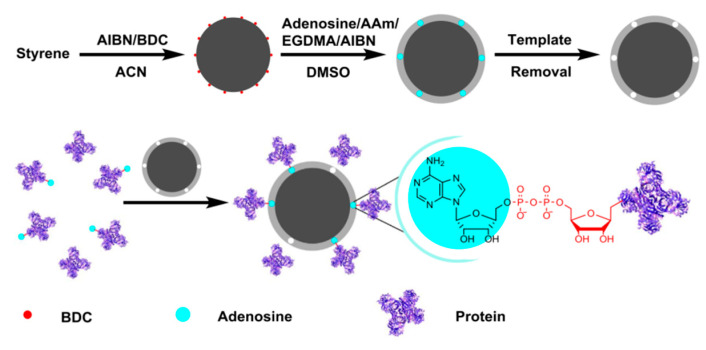
Synthesis route of adenosine-imprinted core-shell microspheres [133].

**Figure 9:**
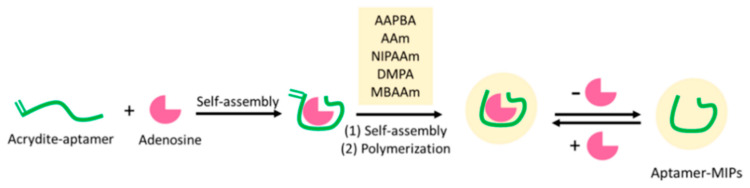
Scheme for preparing aptamer-MIPs [135].

Adsorption is a simple and cost-effective process, which is also easy to regenerate and environmentally friendly. Many researchers have designed and developed a wide range of adsorbents for the specific identification of nucleosides [128,129,130]. However, the conventional adsorbent materials for various nucleoside analogs also have a low adsorption capacity, a slow adsorption rate, and selectivity that needs improvement. Therefore, many researchers have designed and developed a variety of adsorbents for the specific recognition of nucleosides. Their focus includes selecting suitable monomers, optimizing polymerization methods, and creating favorable polymerization environments. These efforts are carried out due to the diverse functional groups present in nucleosides [131,132]. For instance, Xie et al. [133] utilized polystyrene nanoparticles as the substrate material, acrylamide (AM) as the functional monomer, ethylene glycol dimethacrylate (EGDMA) as the cross-linking agent, and adenosine as the template molecule. The adsorbent of adenosine for MIPs was then synthesized via surface-initiated polymerization of the substrates (Figure 8). The resulting imprinted adsorbent exhibited excellent molecular selectivity towards adenosine, with an adsorption capacity of 683 nmol g^−1^, significantly higher than that of guanosine, cytidine, and uridine (91 nmol g^−1^, 24 nmol g^−1^, and 54 nmol g^−1^, respectively). In the research conducted by Krzysztof Szczubiałka’s group [134], silica gel particles were utilized as a substrate material. They utilized dA or 5′-deoxy-5′-(methylthio)adenosine (MTA) as template molecules. Additionally, poly(N-(acryloyloxypropyl)thymine) (MAPIB-APT) and polyanionic (N-(acrylamidooxyethyl)thymine) (AOET-AMPS) were employed as functional monomers. These materials were used to prepare selectively adsorptive materials for discernment imprinting. Liu groups [135] chose 3-acrylamidoethyl adenosine (3-DTA) and 5′-deoxy-5′-(methylthio)adenosine (MTA) as template molecules, while deoxyadenosine does not have a cis-dihydroxyl group. Aptamer-MIPs were prepared using 3-acrylamidophenylboronic acid (AAPBA) as the functional monomer to adsorb adenosine (Figure 9) in this study. The binding between the recognition site and the template molecule was measured by isothermal titration calorimetry (ITC). It was proven that the cis-dihydroxyl of the adenosine molecule showed better affinity for boron. The AAPBA-containing MIPs exhibited a binding capacity for adenosine that was 115 times higher than that of deoxyadenosine and 230 times higher than that of cytidine at pH = 7.6. Table 2 presents a summary of the imprinted polymers’ adsorption capacity, equilibrium time, and ability to selectively identify and separate nucleosides. From the table, it can be seen that different adsorbents have distinct advantages in nucleoside adsorption. However, they still face challenges in terms of low adsorption capacity, slow mass transfer efficiency, and selective identification [124,126,133,136,137,138]. Therefore, a detailed study of efficient adsorbents for the separation of nucleosides is still needed.

## 4. Conclusions and Future Perspective

In summary, different methods for separating nucleoside analogs have both advantages and disadvantages, with adsorption being a widely used, highly efficient, and environmentally friendly method. Although the research on the applications of adsorbents for different nucleoside analogs has a long history, traditional adsorbents also have drawbacks such as low adsorption capacity, slow adsorption rate, and low selectivity. With the continuous breakthroughs in types and quantities of nucleoside analogs, there is also an increasing demand for adsorbents and improved performance requirements. Therefore, achieving the selective separation of nucleosides has become a focal point in research, focusing on the development of new adsorbents with large adsorption capacity, high selectivity, fast adsorption rate, easy regeneration, and good structural stability. The research results play a vital role in promoting the development of antitumor and antiviral drugs. They are also crucial for preventing and controlling serious diseases, as well as enhancing the overall national health.

## Figures and Tables

**Table 1:** Separation method of nucleoside compounds.

Methods	Advantage	Disadvantage	Reference
Crystallization	Inexpensive and easy to industrialize	Toxic solvents or heavy metal salts, environmental pollution, unsuitable for low concentrations products	[82,83,84]
HPLC	Rapid analysis and high-sensitivity	Difficult to implement in industrial applications and high operating costs	[85,86]
Column chromatography	High separation efficiency and simple operation	Organic mobile phase and environmental pollution	[87,88]
Solvent extraction	Large processing capacity and low energy consumption	Organic extraction solvents and selectivity need to be improved	[89,90]
Adsorption	Simple process, inexpensive, easy to regenerate, and environmentally friendly	Selectivity needs to be improved	[91,92,93]

**Table 2:** Comparison of the selectivity adsorption capacities for nucleosides analogs with the other reported MIPs.

Sorbents	Capacity (μmol g^−1^)	Equilibrium (Min)	Imprinting Factor (*IF*)	Reference
J-SNs-MMIPs-Pickering	73.04	60	1.499	[124]
J-MIPs	13.69	240	2.182	[136]
J-SNs-MMIPs	61.22	70	1.570	[137]
MIPs shell	0.363	120	2.011	[133]
J-HNPs-MIPs@Gel	10.31	40	1.730	[126]
ATP-Fe_3_O_4_-NH_2_-DFFPBA	27.17	9	-	[138]

## Data Availability

Not applicable.
